# Long-Term Changes of HIV/AIDS Incidence Rate in China and the U.S. Population From 1994 to 2019: A Join-Point and Age-Period-Cohort Analysis

**DOI:** 10.3389/fpubh.2021.652868

**Published:** 2021-11-15

**Authors:** Yudiyang Ma, Yiran Cui, Qian Hu, Sumaira Mubarik, Donghui Yang, Yuan Jiang, Yifan Yao, Chuanhua Yu

**Affiliations:** ^1^School of Public Health, Department of Epidemiology and Biostatistics, Wuhan University, Wuhan, China; ^2^Department of Public Health, Tongji Medical College, Huazhong University of Science and Technology, Wuhan, China

**Keywords:** HIV, incidence, gender disparities, age-period-cohort effect, trend

## Abstract

Although HIV caused one of the worst epidemics since the late twentieth century, China and the U.S. has made substantial progress to control the spread of HIV/AIDS. However, the trends of HIV/AIDS incidence remain unclear in both countries. Therefore, this study aimed to highlight the long-term trends of HIV/AIDS incidence by gender in China and the U.S. population. The data were retrieved from the Global Burden of Disease (GBD) database since it would be helpful to assess the impact/role of designed policies in the control of HIV/AIDS incidence in both countries. The age-period-cohort (APC) model and join-point regression analysis were employed to estimate the age-period-cohort effect and the average annual percentage change (AAPC) on HIV incidence. Between 1994 and 2019, we observed an oscillating trend of the age-standardized incidence rate (ASIR) in China and an increasing ASIR trend in the U.S. Despite the period effect in China declined for both genders after peaked in 2004, the age effect in China grew among the young (from 15–19 to 25–29) and the old age groups (from 65–69 to 75–79). Similarly, the cohort effect increased among those born in the early (from 1924–1928 to 1934–1938) and the latest birth groups (from 1979–1983 to 2004–2009). In the case of the U.S., the age effect declined after it peaked in the 25–29 age group. People born in recent birth groups had a higher cohort effect than those born in early groups. In both countries, women were less infected by HIV than men. Therefore, besides effective strategies and awareness essential to protect the young age groups from HIV risk factors, the Chinese government should pay attention to the elderly who lacked family support and were exposed to HIV risk factors.

## Introduction

Human immunodeficiency virus and/or Acquired Immune Deficiency Syndrome are both severe sexually transmitted diseases. HIV/AIDS is no more than a public health issue but a social problem (1). Four decades after its emergence, HIV remains dominant among sexually transmitted diseases that threaten the health of people worldwide (2, 3). According to an Global Burden of Disease Study (GBD) (4), HIV leads to various opportunistic infections. Despite advanced in medicine, the disease burden of ADIS remained severe. HIV also causes much of health care costs lost due to it is an incurable disease until now. The most common therapeutic regimes, such as antiretroviral therapy (ART), were only effective at durably suppressing the replication of HIV and improving survival but cannot eliminate the virus and cure AIDS (5, 6).

The HIV epidemic in China originated from injection in drug users (IDU). Then, HIV propagated from Yunnan Province to other Chinese regions along the drug trafficking routes in the mid-1990s (7). With the development of society, the patterns of transmission evolved because injecting drugs or sharing needles for blood transfusion were banned by the law in the mid and late 1990s (8). Sexual transmission has become the major route of HIV infection in China (9). According to recent studies (10), the rising part of newly confirmed patients ascribed to the sexual transmission had increased from 11% in 2005 to 96% in 2017. The heterosexual contact transmission rose from 11 to 70%, while the homosexual contact transmission rose from nearly 0–26%. Similarly, sexual transmission was common in the U.S. The incidence decreased for each transmission risk category except for the sexual transmission from 2008 to 2015 (11).

With its immense population and as the largest developing country in the world, minds, economy, lifestyle, even attitudes to sex in Chinese citizens were gradually close to the U.S. (12). Men who have sex with men (MSM), heterosexuals, and IDU were the three main transmission routes of HIV infections in China and the U.S. More significantly, guidance for the HIV epidemic control efforts in China may be gained from the experience of the U.S. (13). In addition, the study of Gao et al. (14) found that the risk of HIV mortality in China rapidly increases and Liu et al. (15) denoted existing great differences in the HIV incidence between men and women. Under this background, understanding the temporal trends of HIV incidence through the demographic lens on the national level enables us to examine whether the epidemic of HIV change at different rates for different age or cohort groups during the same period. Independent effects of age, period, birth cohort and temporal trends were analyzed by the age-period-cohort model (APC) with an intrinsic estimator (IE) algorithm and Join-point regression, using data from the GBD study 2019. By using the updated GBD data, our study aimed to investigate the long-term trends of AIDS incidence by gender in China and the U.S. during 1994–2019.

## Methods

### Data Sources

Contributed by the Institute for Health Metrics and Evaluation (IHME; http://www.healthdata.org/gbd/2019), the GBD 2019 aims to quantify the comparative magnitude of health loss due to diseases, injuries, and risk factors by age, sex, and geographies for specific points in a series of times. Estimates such as incidence, death, and prevalence were conducted among groups stratified by age, sex, year, and location on the GBD (16). All anonymized data have been publicly available at the IHME website and can be accessed online (http://ghdx.healthdata.org/). HIV/AIDS events were diagnosed and classified based on the WHO clinical criteria, the International Statistical Classification of Diseases, and the International Classification of Diseases and Injuries (ICD-10). For HIV, ICD-10 codes are B20-B24, C46-C469, D84.9. Generally, household surveys with complete summary birth histories, censuses, disease surveillance system, vital registration, and sample registration systems (from the Disease Surveillance Point system and the Notifiable Infectious Disease Reporting system, which were administered by the Chinese Center for Disease Control and Prevention) constitute the primary data input for the GBD (3). The modeling strategy for estimating HIV incidence was generalized by grouping countries based on quality and types of data available (17). Our data on HIV/AIDS were obtained from the global health data exchange (GHDx) section result tools (http://ghdx.healthdata.org/gbd-results-tool). Ethical approval was not needed for this study because there was no direct involvement of human subjects.

### Statistical Analysis

#### Age–Period–Cohort Analysis and Join-Point Regression Analysis

The APC is a prevalently statistical tool to extract information hidden in age-adjusted incidence. It is used for long-term trend studies such as social changes, disease causes, aging, population process, and dynamic research (18). The age effect indicates the different risks of various outcomes during distinct periods of life; the period effect reflects population-wide exposure at a circumscribed point in time; the cohort effect generally represents the disparities of risk across birth cohorts (19). Collinearity problem caused by the linear relationship between age, period, and cohort can be further expressed as *cohort* = *period*−*age* (20). To address this identification problem, the IE algorithm was used to solve the APC problem. The APC model with an IE algorithm has been proved to be unbiased, estimable, and valid in solving the identification problem (18). Therefore, we conducted the IE to calculate the coefficient.

The annual percentage change (APC) and the average annual percentage change (AAPC) for each segment were estimated by Join-point regression which focused on estimating the temporal trends in the age-standardized incidence rate (ASIR) of HIV. A maximum number of four points were allowed, ensuring the results were credible. We applied the Join-point Regression program version 4.8.0.1 (Information Management Services Inc., Calverton, MD, USA) from the Statistical Research and Applications Branch of the Surveillance Research Program of the U.S. National Cancer Institute.

#### Data Arrangement

The time series from 1994 to 2019 was chopped into five consecutive intervals. The data before 1994 were discarded because from 1990 to 1993 was not a continuous 5-year interval. Successive 5-year age groups from 15–19 to 75–79 years were divided. Individuals younger than 15 and older than 80 were ruled out. Based on the WHO report (21), the population was classified into young (age <44 years), middle (age 45–59 years), and old age groups (age > 60 years). Besides these, 18 consecutive cohorts were allotted, including those born from 1919–1923 to 2004–2009. The estimated coefficients for the age, period, and cohort effects by APC analysis were in Supplementary Tables 2, 3. The incidence risk ratio (RR) was the exponential value of the coefficient, which denoted the risk of infection in particular age, period, or birth cohort relative to the reference groups. The mean level of age, period, and cohort was selected as reference groups. All analyses were conducted by Stata 14 software (StataCorp, College Station, TX, USA). The Wald chi-square test was utilized to calculate the significance of the estimable parameters and functions. All statistical tests were 2-sided and the significance level considered was 5%. Model fitting was evaluated by deviance, SE coefficients, Akaike's information criterion (AIC), and the Bayesian information criterion (BIC).

## Results

### The HIV/AIDS Incidence Trend in China and the U.S

**Figure 1:**
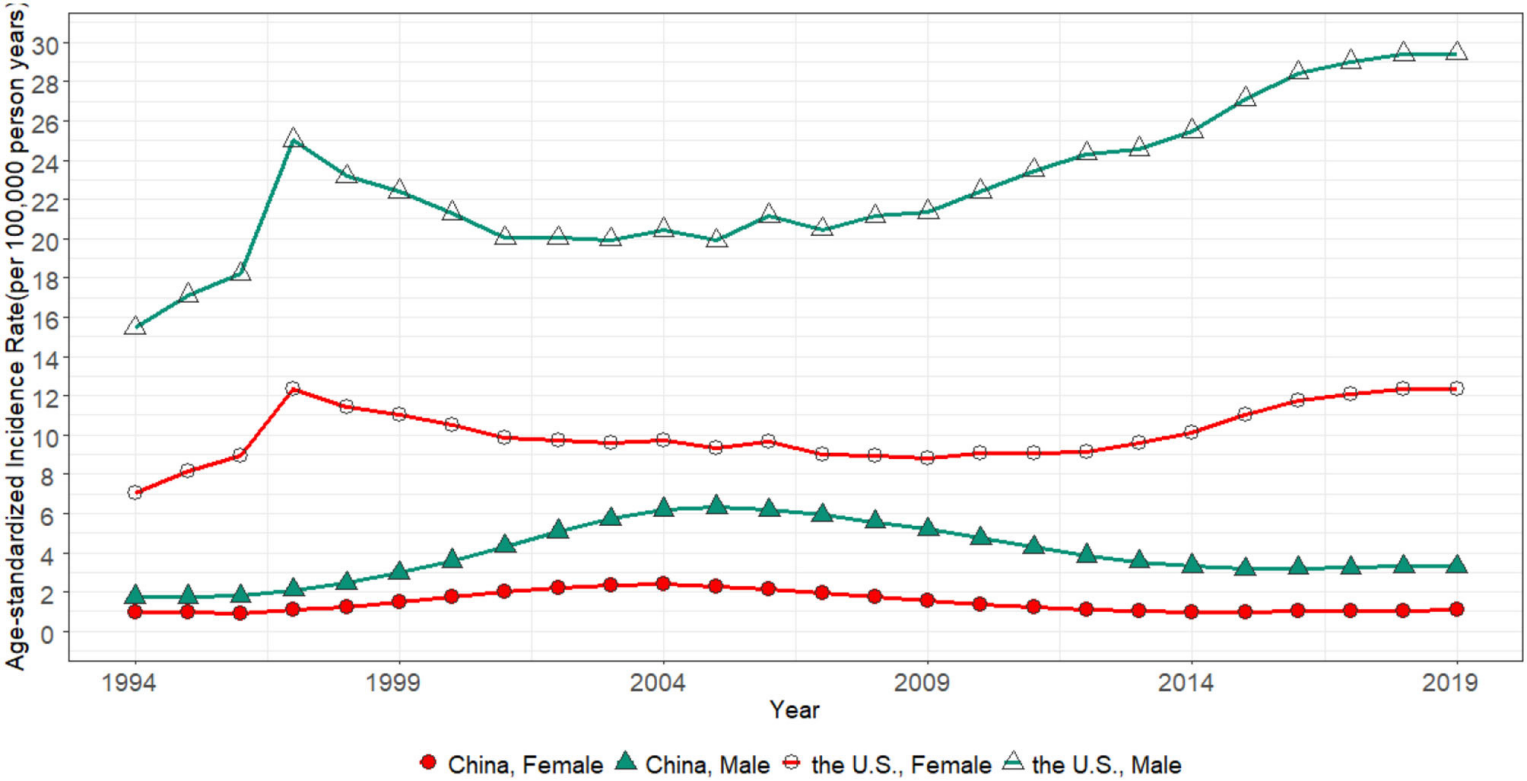
The age-standardized incidence rate for China and the U.S. from 1994 to 2019.

Figure 1 presents the trend of the ASIR for HIV/AIDS in China and the U.S. from 1994 to 2019. The ASIR of men was invariably higher than women in both countries. In the U.S., the ASIR peak in 1997, while it gradually grew up from 2001 to 2019. While in the men of China, the ASIR rose from 1.76/100,000 to 6.34/100,000 in 2005, then it dropped from 6.19/100,000 to 3.30/100,000 in 2019. On the other hand, the ASIR of women was kept low, wherein the range of changes from 1994 to 2019 did not exceed 1.5/100,000.

Table 1 presents the APC and AAPC of HIV incidence in China and the U.S. from 1994 to 2019. In men of China, the ASIR increased from 1994 to 2006 then decreased from 2007 to 2019, whereas women of China had a similar change. The AAPC in the whole period was 2.6% (1.9 and 3.4%) and 0.3% (0.1 and 0.6%) for men and women, respectively. In the U.S., the ASIR kept increasing in recent years, with an overall AAPC value of 2.8% (2.1 and 3.5%) for men and 2.4% (1.6 and 3.2%) for women. For age-specific rates, incidence increased almost all age groups in men of China and both genders of the U.S. The incidence declined in most age groups except for those aged 15–25 or 60–69 years in women (Supplementary Table 1).

**Table 1:** Trends in HIV ASIR by gender in China and the U.S., 1994–2019.

**Segments**	**Year**	**Male**			**Female**		
		APC*	**95%CI**		APC*	**95%CI**	
			**lower**	**upper**		**lower**	**upper**
**China**							
Trend1	1994–1996	0.5	−4.4	5.7	−3.0	−6.4	0.5
Trend2	1996–2003	18.9*	17.8	20.1	16.7*	15.7	17.6
Trend3	2003–2006	2.6	−1.9	7.3	0.5	−2.4	3.6
Trend4	2006–2015	−7.8*	−8.2	−7.3	−10.1*	−10.4	−9.7
Trend5	2015–2019	1.7	−0.3	3.8	0.7*	0.1	1.4
AAPC^a^	1990–2019	2.6*	1.9	3.4	0.3*	0.0	0.8
**The U.S**.							
Trend1	1994–1996	17.0*	14.1	19.8	20.0*	16.6	23.6
Trend2	1996–2003	−3.6*	−4.9	−2.2	−4.3*	−6.8	−1.7
Trend3	2003–2006	1.2*	0.3	2.0	−1.2*	−1.7	−0.7
Trend4	2006–2015	3.9*	3.1	4.8	5.6*	4.0	7.2
Trend5	2015–2019	0.5	−5.7	7.1	1.0	−6.2	8.6
AAPC^b^	1990–2019	2.8*	2.1	3.5	2.4*	1.6	3.2

a *APC, annual percentage change*;

b *AAPC, average annual percent change; CI, confidence interval*;

* *Significantly different from 0 at alpha = 0.05 (p < 0.05)*.

### Gender Disparities for Men and Women in Both Countries

The difference in the ASIR between men and women can be observed in Figure 1. In China, it expanded from 0.78/100,000 in 1994 to 4.07/100,000 in 2006, but it had shrunk from 3.99/100,000 in 2007 to 2.23/100,000 in 2019. A considerable gender disparity on the ASIR existed in the U.S. and increased from 8.38/100,000 in 1994 to 17.08/100,000 in 2019. The gap possibly narrowed from 1997 to 2001. This promising trend did not keep hold.

**Figure 2:**
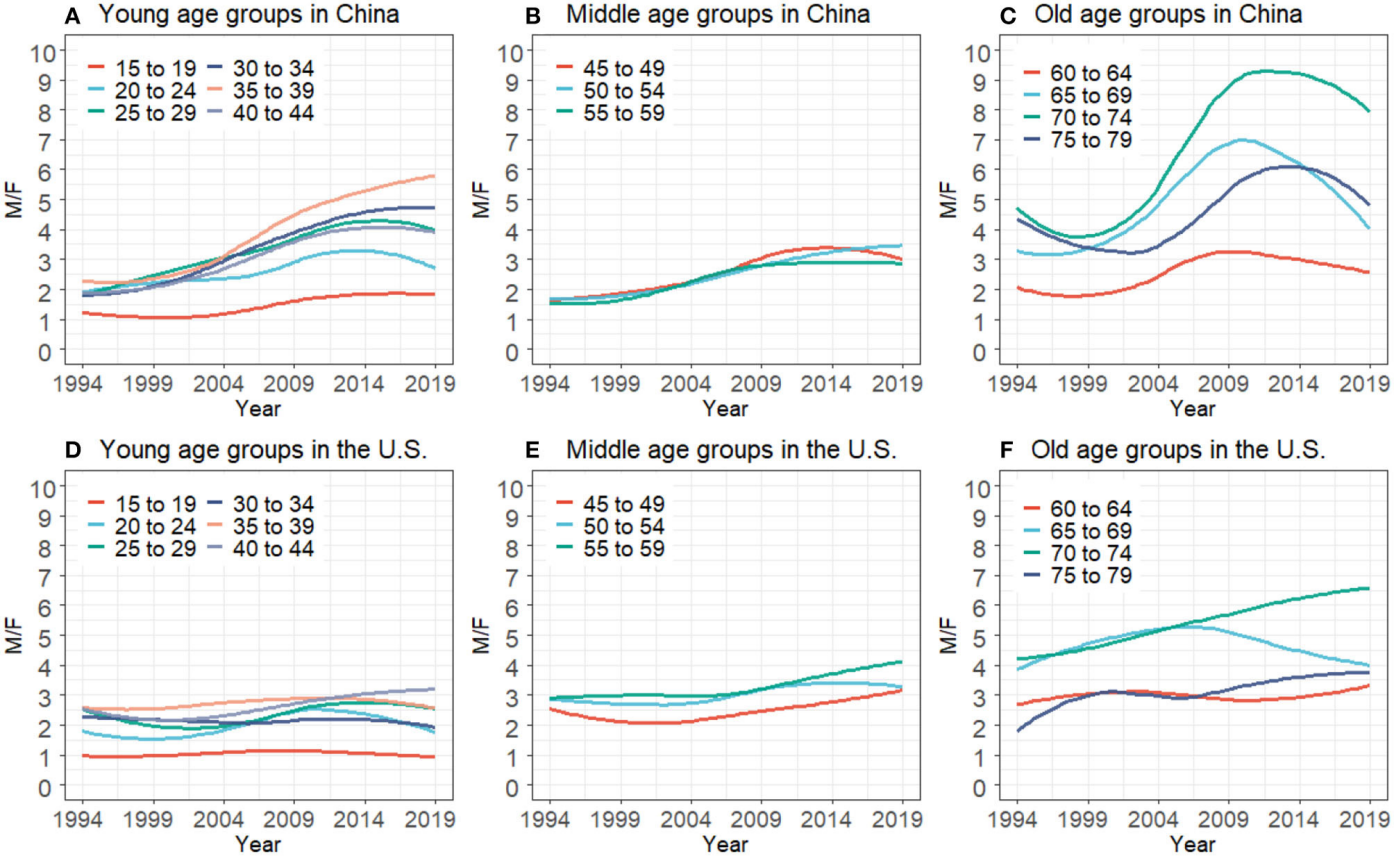
HIV/AIDS men-to-women incidence ratios for the young, middle, and old age groups in China **(A–C)** and the U.S. **(D–F)** from 1994 to 2019.

Figure 2 demonstrates the men-to-women incidence ratio, wherein an index >1 indicates the HIV incidence is higher in men than women. In the U.S., compared with the old age groups (Figure 2F), ratios at young age groups (Figure 2D) in different periods showed slight growth. In China, men-to-women incidence ratios in young age groups (Figure 2A) increased from 1994 to 2019. Although ratios in middle age groups (Figure 2B) were stable at a low level, ratios in old age groups (Figure 2C) presented noticeable fluctuations. However, men-to-women incidence ratios only reflected trends of gender disparities in HIV incidence with time or age change, which might cover the severity of HIV/AIDS incidence to some degree.

### Age Effect

**Figure 3:**
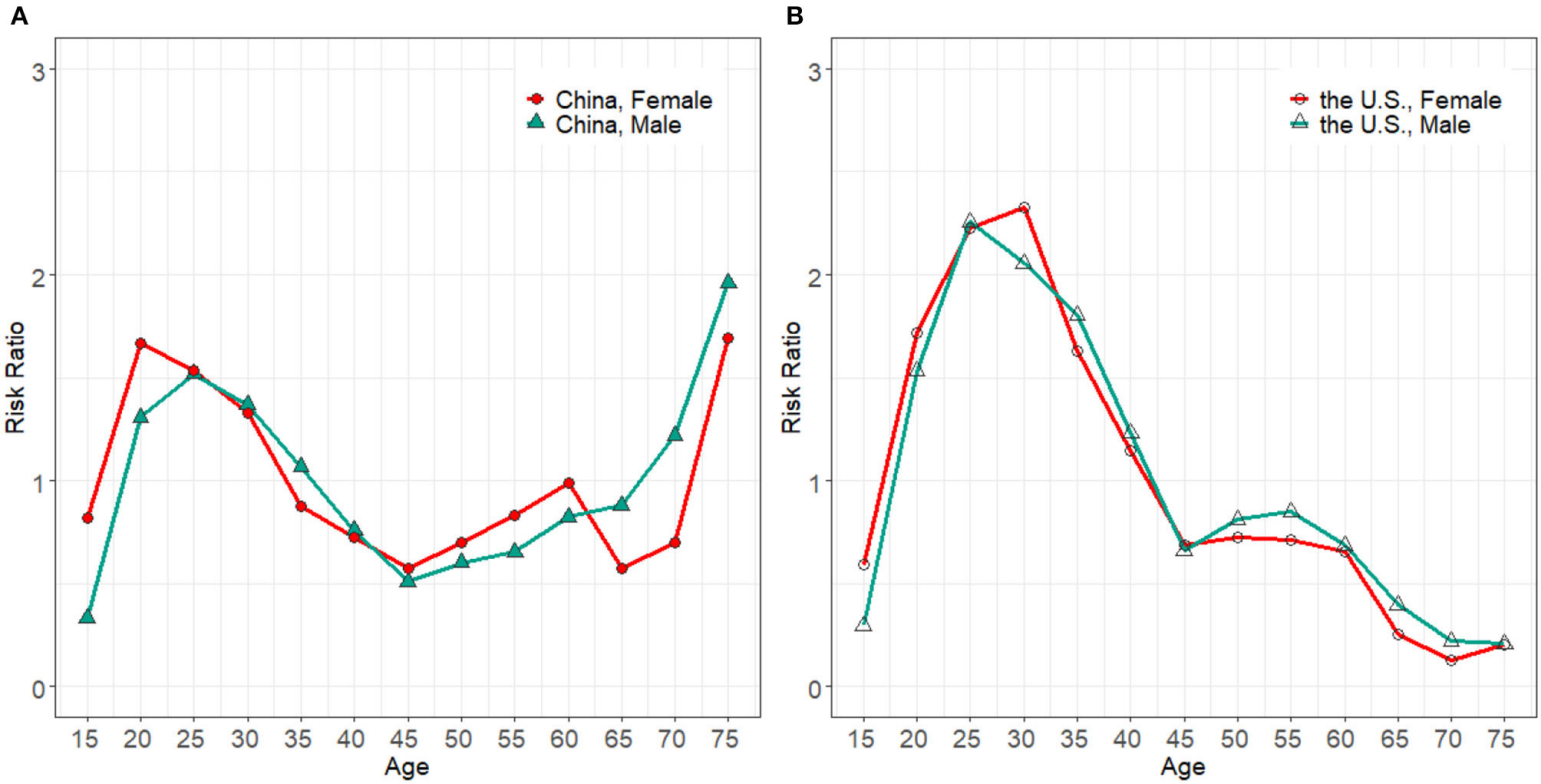
Age effect on HIV/AIDS incidence for males and females in China **(A)** and the U.S. **(B)**, from 1994 to 2019.

The age effect (Figure 3) rose from the 15–19 age group quickly and peaked in the 25–29 age group. Then, it glided down in middle-aged groups from 30–34 to 45–49 age groups. The trend of age effect for the young and middle age groups was resemblant in both countries. At the 25–29 age groups, the RR in the U.S. was 1.5 and 1.4 times larger than in China for men and women. The age effect of both genders in the U.S. glided down to a rock-bottom position in the 75–79 age group, but for China, an opposite trend was observed. The age effect exhibited an increasing trend from 45–49 to 75–79 age groups in males of China and reached the highest in the 75–79 age group, in which the RR were 9.5 and 8.4 times larger than men and women in the U.S., respectively.

### Period Effect

**Figure 4:**
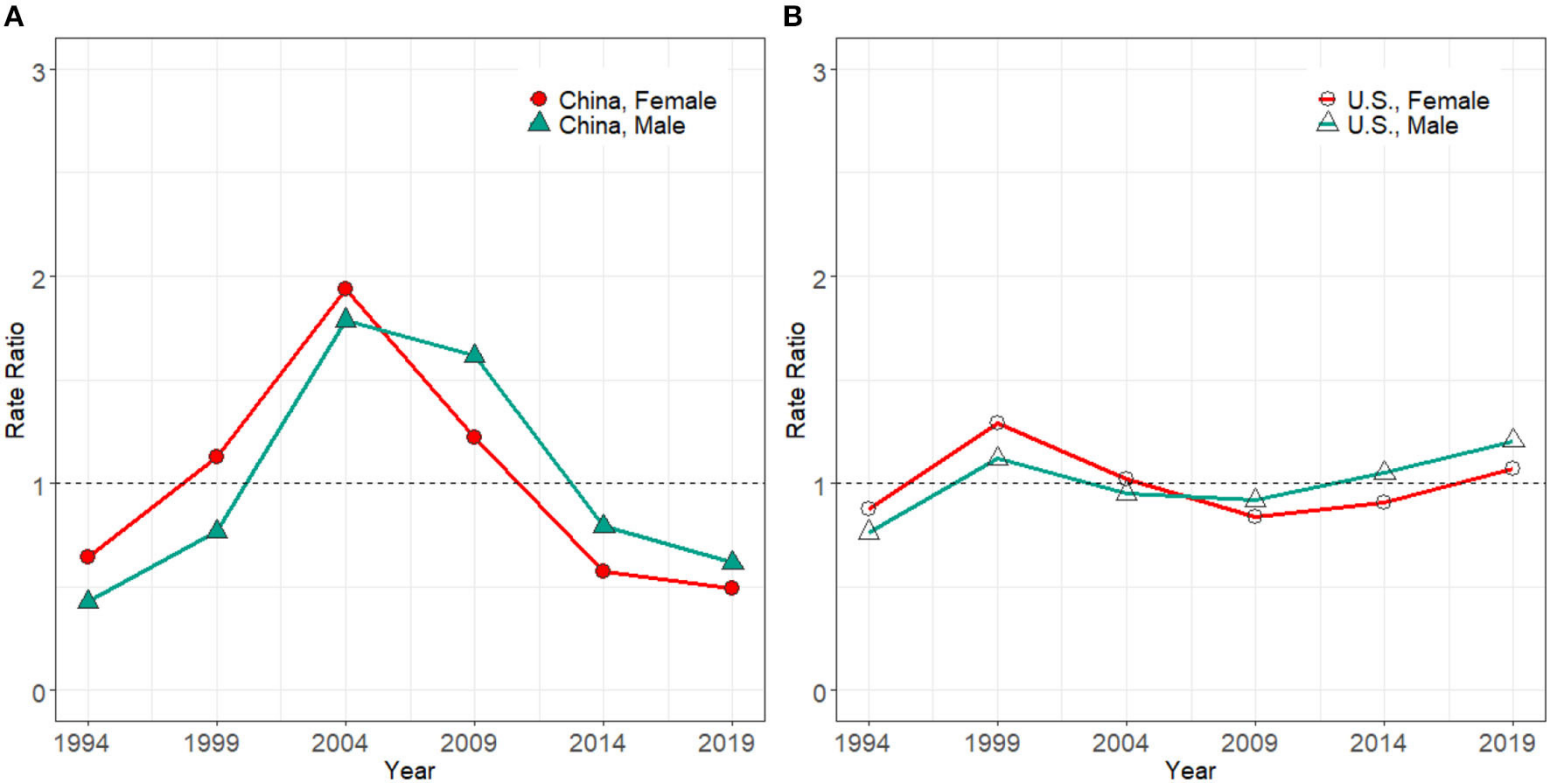
Period effect on HIV/AIDS incidence rate for male and females in China **(A)** and the U.S. **(B)**, from 1994 to 2019.

The period effect in the U.S. fluctuated modestly as shown in Figure 4B. Compared with the RR in 2009, it just increased by 1.6 and 1.2 times in respective men and women in 2019. The trend of period effect in China oscillated more strikingly (Figure 4A). It had no sign of decline until it reached the highest in 2004. The RR for both men and women in China in 2014 was, respectively, 1.87 and 1.9 times larger than that of the U.S. during the same period. After which, the upward momentum was curbed and glided down to the level of 1994.

### Birth Cohort Effect

**Figure 5:**
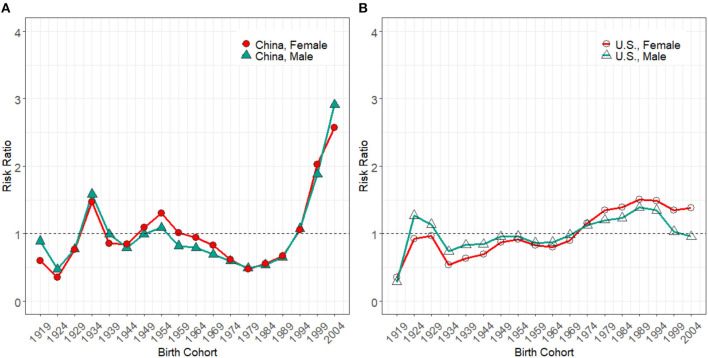
Cohort effect on HIV/AIDS incidence rate for male and female in China **(A)** and the U.S. **(B)** from 1994 to 2019.

The cohort effect (Figure 5B) in the U.S. grew gradually from the early to the latest birth cohort. It increased by 238.2% in men and 290.1% in women. Compared with the U.S., the cohort effect seemed more complex in China (Figure 5A). The cohort effect presented an up and down trend for those born between 1919–1923 and 1979–1983 birth groups. Then a rapid growth was seen from 1979–1983 to 2004–2009 birth groups, with the increments of 227.6% in men and 329.0% in women in China. In both countries, the recent birth groups were exposed to relatively higher HIV/AIDS incidence risks than earlier birth groups.

The RR and the corresponding coefficients of age, period, and cohort estimated by the APC model are presented in Supplementary Tables 2, 3.

## Discussions

Our study explored the temporal trends in HIV incidence with the aid of the APC model with an IE algorithm and Join-point regression analysis. Between 1994 and 2019, we observed an oscillating trend of the ASIR in China and an increasing ASIR trend in the U.S. The age and cohort effect in the U.S. indicated that the younger generations had a higher risk of HIV infection than other groups. However, besides the younger generations, the older generations in China were also at a high risk of HIV infection. For the gender disparities of HIV incidence in China and the U.S., it can be attributed to the following reasons.

First, based on the World Health Statistics Annual (22–24), there existed a significant gender gap in China and the U.S. from 1994 to 2019. The gap may cause a higher HIV incidence among males who are more likely to have high-risk HIV behaviors such as inconsistent condom use, having multiple sexual partners, or alcohol/drug abuse (25). Another was that MSM became popular. Chinese sexual liberation freed the minds of people and the legalization of homosexuality in 1997 (7). The MSM infection in China increased from almost 0% in 2005 to 26% in 2017. Whereas in the U.S., MSM accounted for 70% of new HIV diagnoses in 2017 (10, 26). Therefore, the number of infected men was higher than the number of infected women.

Human immunodeficiency virus infections in the young proved an issue that should be addressed carefully in both countries. Young age groups born in recent birth cohorts enjoyed much greater freedoms in thought, speech, and choices. The setting change made considerable biological, physiologic even mental changes in them (27), so they were easier to get hooked on some common risk factors of AIDS and bear a high risk of infection. The elderly born earlier in China faced severe HIV infections, while the U.S. was free of this problem. Chinese residency system reform in 1980 compelled millions of older migrants to leave their native places (28). Education and expertise scarce compelled them away from home to earn a living. Lack of family support might generate influences on opportunistic infections among the old generation (29). Comparing with non-migrants living with families, elderly migrants were far away from home and may seek sexual services. Unprotected sexual intercourse was common in these groups due to short of education and out-of-pocket (30). Hence, sexually risky behaviors caused an exacerbation of the HIV/AIDS epidemic in older groups. Besides, the sexual demands of the elders were often neglected by their partners as well as by society (31). For the sake of dispelling loneliness, they chatted on the internet *via* various kinds of mobile applications and dated with unfamiliar persons without sufficient background information (32). Short of self-protection consciousness among older adults eventually increased the risk of HIV/AIDS acquisition. If these issues get no attention from the government, acute aging problems in China (33) may intensify the current HIV/AIDS incidence situation and lead to a new wave of increasing.

The period effect changed modestly in the U.S. when cohort and age effects were controlled. This could be attributed to advanced healthcare systems (34) and developed AIDS surveillance in the U.S. (35). Nevertheless, therapies were only effective at durably suppressing the replication of HIV but could not eliminate the virus (36), and uncontrollable high-risk behaviors (37) may cause HIV resurgence. At the same time, some aggressive treatment therapies accompanied with side-effects too (24). Suffering severe side-effects made the patients hesitate to accept therapies that will cause HIV resurgence.

For China, the period effect was consistent with the ASIR. According to former studies (38, 39), the time before 2005 was described as the “rapid expansion phase.” The upward tendency of the ASIR could be the reform and opening-up policy. Before that, traditional Chinese held that premarital or extramarital sex was shameful, and their attitudes on sex were limited to marital sex, with the purpose of reproductions (40). The introduction of “the Open Door Policy” brought traditional Chinese people to accept pre- and extra-marital sex widely (41, 42). The turning point started in 2003 when the “Four Frees and One Care” policy was announced by the Chinese government (43). Then in 2005, a web-based HIV reporting system integrated with HIV/AIDS surveillance system was established (44). Chinese Center for Disease Control and Prevention launched a web-based HIV/AIDS information system and the Comprehensive Response Information Management System in 2006 (44). The country delivered health promotion strategies in social networks, strengthening sexual health services and partner services (45, 46), and these measures made a breakthrough contribution to curbing HIV.

A previous study (47) using data from the National Comprehensive AIDS Prevention and Control Information System in Zhejiang province indicated that the higher AIDS incidence risks were distributed in young people. It was consistent with our analysis. However, their research lacked representation of long-term HIV trends in China, such as ignoring the incidence of AIDS among the elderly, because they focused on just one province in China in a shorter period. In addition, the IE algorithm in our study was used to get the net effects for the three different temporal dimensions and address the identification problem (18, 48). Likewise, the studies of Huang et al. and Segarra et al. (49, 50) described the trends of the AIDS incidence in China and the U.S. without the APC model, which might miss the information hidden in age-adjusted AIDS incidence. Compared with the above research, the advantages of our study were more comprehensive and representative. Based on the latest GBD 2019 study, this study found a rapidly increasing incidence of AIDS in the U.S. and complex epidemic status in China. The age effect associated with behavior changes throughout the life cycle of a human might be the most key factor affecting HIV incidence in the population. Young people were at increased risk of infecting HIV from the age of 15 onward. Among the young age group, the risk of HIV infection was highest in the 20–30 age group in China or the 25–35 age group in the U.S. The risk for HIV infection also increased in the elderly in China and people aged 80–84 years were at the highest risk for infection. The period effect was linked to medical progress and policymaking, which influenced the overall change of HIV incidence. The cohort effect was related to the birth environment or background, so those born recently were at high risk of HIV infection in both countries. Therefore, it is necessary to develop the strategies according to the different backgrounds of birth or different age stage to reduce the AIDS incidence in China and the U.S.

## Limitation

There were several limitations to this study. First, although the GBD 2019 adapted numerous adjustments and corrections to the source, collation, and evaluation of the HIV/AIDS incidence to enhance data accuracy and comparability, it was undeniable that certain deviations in the completeness and accuracy of the GBD data were inevitable, such as the estimation of incidence and mortality data on the U.S. (51). Second, our study lacked the analysis of comparison of HIV incidence in the different provinces because of the unavailability of data. Third, similar to other studies based on a population level, the APC-IE framework concluded the results at the population level. Ecological fallacy might occur because the study might not focus on the individual level. Therefore, more relevant studies focusing on these limitations should be carried out in the future to reveal more accurate analysis results.

## Conclusions

In summary, our study estimated the long-term trends of HIV incidence by gender in China and the U.S. from 1994 to 2019. We found that the ASIR of HIV for both men and women in China kept on the same decreasing track recently. At the same time, it grew yearly for both genders in the U.S. By APC analysis, the younger generations, especially in their 20–35 years, had a high risk of HIV infection in China and the U.S., but the infection risk for the young in the U.S. was higher than China. Despite the period effect decline continuously, it was worth noting that the HIV infection risk in the Chinese elderly increased with age. Men were more threatened by HIV than women. Therefore, besides effective strategies and awareness essential to protect the young age groups from HIV risk factors, the Chinese government should pay attention to the elderly who lacked family support and were exposed to HIV risk factors.

## Data Availability Statement

The datasets generated and analyzed during the current study are available in the GBD results tool repository (http://ghdx.healthdata.org/gbd-results-tool).

## Author Contributions

YM designed the study, implemented the study protocol, collected and analyzed data, and wrote the first manuscript. YC, YJ, and YY directed statistical analyses of the data and revised the paper. QH analyzed and interpreted the data. CY, SM, and DY revised the manuscript. All authors approved the final manuscript.

## Funding

This study was funded by the National Key Research and Development Program of China [grant numbers 2018YFC1315302, 2017YFC1200502] and the National Natural Science Foundation of China [grant number 81773552].

## Conflict of Interest

The authors declare that the research was conducted in the absence of any commercial or financial relationships that could be construed as a potential conflict of interest.

## Publisher's Note

All claims expressed in this article are solely those of the authors and do not necessarily represent those of their affiliated organizations, or those of the publisher, the editors and the reviewers. Any product that may be evaluated in this article, or claim that may be made by its manufacturer, is not guaranteed or endorsed by the publisher.
