# Supplementary material for: Long-Term Changes of HIV/AIDS Incidence Rate in China and the U.S. Population From 1994 to 2019: A Join-Point and Age-Period-Cohort Analysis
Source: Front Public Health. 2021 Nov 15;9:652868. doi: 10.3389/fpubh.2021.652868 (PMC8634360; doi:10.3389/fpubh.2021.652868)
Supplement: Supplementary file 1 [file Data_Sheet_1.PDF]

Supplementary Material

Long-Term Changes of HIV/AIDS Incidence Rate in China and the U.S. Population From 1994-2019: A Jointpoint and Age-Period-Cohort analysis

Supplementary Figures and Tables

1.1 Supplementary Tables

Supplementary Table 1. The average annual percent changes (AAPCs) in HIV incidence of male and female in China and the U.S., 1994–2019.

Age group	China (AAPC)		the U.S. (AAPC)	
	Male	Female	Male	Female
15-19	7.2(5.9, 8.5)	4.9(3.8, 5.9)	-0.4(-2.5, 1.8)	-0.2(-1.2, 0.8)
20-24	5.5(4.7, 6.4)	3.8(2.9, 4.8)	0.8(0.0, 1.60)	0.5(0.1, 0.6)
25-29	2.7(2.2, 3.3)	-0.4(-1.2, 0.3)	3.1(2.1, 4.1)	2.9(1.2, 4.6)
30-34	1.3(0.9, 1.7)	-2.6(-3.1, -2.0)	3.5(2.5, 4.5)	4.1(3.3, 5.0)
35-39	1.1(0.7, 1.4)	-3.1(-4.8, -1.3)	4.0(2.9, 5.1)	4.4(3.0, 5.7)
40-44	0.0(-0.3, 0.3)	-3.2(-3.9, -2.5)	4.3(3.4, 5.1)	3.4(2.3, 4.5)
45-49	0.6(-0.3, 1.4)	-2.0(-2.8, -1.1)	2.9(1.7, 4.1)	1.8(0.3, 3.3)
50-54	1.3(0.3, 2.4)	-1.5(-2.8, -0.3)	1.1(0.0, 2.2)	0.2(-1.5, 2.1)
55-59	1.8(0.9, 2.7)	-0.7(-1.9, 0.4)	3.2(2.5, 4.3)	2.2(1.2, 3.1)
60-64	2.4(1.6, 3.1)	0.9(0.2, 1.6)	4.3(3.1, 5.5)	3.4(2.3, 4.6)
65-69	3.2(2.5, 3.9)	1,7(0.9, 2.5)	4.8(3.2, 6.4)	4.7(3.5, 5.9)
70-74	1.5(0.6, 2.3)	-1.3(-2.2, -0.4)	0.6(-0.7, 1.9)	-1.2(-2.6, 0.2)
75-79	-1.0(3.3, 1.4)	-2.5(-4.0, -0.9)	3.5(0.1, 8.2)	0.2(-4.2, 4.7)
**Age-standardized Rate**	2.6(1.9, 2.4)	0.3(0.0, 0.8)	2.8(2.1, 3.5)	2.4(1.6, 3.2)

Supplementary Table 2. The relative ratio of HIV/AIDS incidence due to age, period, and cohort effects in China,1994 to 2019

APC Factor	Males				Females			
	Coef.	RR	95%CI		Coef.	RR	95%CI	
			Lower	Upper			Lower	Upper
**Age**								
15-19	-0.99	0.33	0.21	0.65	-0.13	0.82	0.42	1.82
20-24	0.37	1.31	1.03	2.05	0.58	1.67	1.02	3.16

25-29	0.52	1.52	1.2	2.39	0.5	1.53	0.9	3
30-34	0.42	1.37	1.05	2.2	0.36	1.33	0.76	2.66
35-39	0.17	1.07	0.79	1.77	-0.06	0.88	0.46	1.92
40-44	-0.17	0.76	0.54	1.31	-0.26	0.72	0.38	1.6
45-49	-0.57	0.51	0.35	0.92	-0.48	0.58	0.29	1.32
50-54	-0.41	0.6	0.43	1.03	-0.28	0.7	0.38	1.49
55-59	-0.32	0.65	0.48	1.09	-0.11	0.83	0.47	1.7
60-64	-0.09	0.82	0.64	1.31	0.06	0.99	0.6	1.9
65-69	-0.02	0.88	0.7	1.36	-0.48	0.58	0.31	1.25
70-74	0.3	1.22	1	1.84	-0.29	0.7	0.38	1.46
75-79	0.78	1.96	1.62	2.94	0.6	1.69	1.01	3.25
**Period**								
1994	-0.72	0.43	0.35	0.66	-0.32	0.64	0.46	1.15
1999	-0.14	0.77	0.68	1.13	0.24	1.13	0.86	1.88
2004	0.71	1.79	1.68	2.46	0.78	1.94	1.57	3.03
2009	0.61	1.61	1.52	2.23	0.32	1.22	0.95	1.97
2014	-0.1	0.79	0.7	1.15	-0.43	0.58	0.41	1.03
2019	-0.36	0.61	0.53	0.92	-0.59	0.49	0.33	0.94
**Birth Cohort**								
1919-1923	0	0.89	0.44	2.27	-0.39	0.6	0.15	3.06
1924-1928	-0.64	0.47	0.26	1.08	-0.93	0.35	0.1	1.54
1929-1933	-0.14	0.77	0.57	1.34	-0.14	0.77	0.39	1.93
1934-1938	0.58	1.58	1.31	2.44	0.51	1.47	0.9	3.03
1939-1943	0.11	0.99	0.79	1.58	-0.04	0.85	0.47	1.95
1944-1948	-0.11	0.79	0.61	1.3	-0.04	0.85	0.48	1.92
1949-1953	0.12	1	0.76	1.68	0.21	1.09	0.6	2.51
1954-1958	0.2	1.09	0.8	1.88	0.39	1.3	0.73	3
1959-1963	-0.08	0.82	0.57	1.49	0.13	1.01	0.53	2.46
1964-1968	-0.12	0.79	0.56	1.42	0.06	0.94	0.5	2.25
1969-1973	-0.25	0.69	0.5	1.22	-0.07	0.82	0.45	1.92
1974-1978	-0.4	0.59	0.44	1.02	-0.37	0.61	0.34	1.4
1979-1983	-0.6	0.49	0.36	0.84	-0.62	0.48	0.26	1.11
1984-1988	-0.51	0.53	0.4	0.9	-0.47	0.55	0.32	1.23
1989-1993	-0.31	0.65	0.48	1.11	-0.28	0.67	0.38	1.51
1994-1998	0.19	1.07	0.78	1.88	0.17	1.05	0.56	2.53
1999-2003	0.75	1.88	1.23	3.63	0.83	2.02	0.97	5.37
2004-2009	1.19	2.91	1.09	9.87	1.07	2.57	0.63	7.33
**AIC**		4.47				3.31		
**BIC**		-176.63				-187.98		
**Deviance**		15.06				3.71		

Supplementary Table 3. The relative ratio of HIV/AIDS incidence due to age, period, and cohort effects in U.S.,1994 to 2019

**APC Factor**	**Males**				**Females**			
	**Coef.**	**RR**	**95%CI**		**Coef.**	**RR**	**95%CI**	
			**Lower**	**Upper**			**Lower**	**Upper**

**Age**								
15-19	-0.93	0.3	0.19	0.65	-0.13	0.82	0.42	1.82
20-24	0.71	1.53	1.28	2.05	0.58	1.67	1.02	3.16
25-29	1.1	2.26	1.76	2.39	0.5	1.53	0.9	3
30-34	1	2.05	1.56	2.2	0.36	1.33	0.76	2.66
35-39	0.87	1.8	1.38	1.77	-0.06	0.88	0.46	1.92
40-44	0.49	1.23	0.93	1.31	-0.26	0.72	0.38	1.6
45-49	-0.13	0.66	0.47	0.92	-0.48	0.58	0.29	1.32
50-54	0.07	0.81	0.68	1.03	-0.28	0.7	0.38	1.49
55-59	0.12	0.85	0.71	1.09	-0.11	0.83	0.47	1.7
60-64	-0.1	0.68	0.49	1.31	0.06	0.99	0.6	1.9
65-69	-0.64	0.4	0.29	1.36	-0.48	0.58	0.31	1.25
70-74	-1.24	0.22	0.12	1.84	-0.29	0.7	0.38	1.46
75-79	-1.3	0.21	0.11	2.94	0.6	1.69	1.01	3.25
**Period**								
1994	-0.26	0.76	0.67	0.66	-0.32	0.64	0.46	1.15
1999	0.12	1.12	1.01	1.13	0.24	1.13	0.86	1.88
2004	-0.04	0.95	0.87	2.46	0.78	1.94	1.57	3.03
2009	-0.08	0.92	0.84	2.23	0.32	1.22	0.95	1.97
2014	0.06	1.05	0.95	1.15	-0.43	0.58	0.41	1.03
2019	0.2	1.2	1.07	0.92	-0.59	0.49	0.33	0.94
**Birth Cohort**								
1919-1923	-1.22	0.28	0.06	2.27	-0.39	0.6	0.15	3.06
1924-1928	0.28	1.27	0.73	1.08	-0.93	0.35	0.1	1.54
1929-1933	0.17	1.14	0.68	1.34	-0.14	0.77	0.39	1.93
1934-1938	-0.26	0.74	0.49	2.44	0.51	1.47	0.9	3.03
1939-1943	-0.14	0.83	0.6	1.58	-0.04	0.85	0.47	1.95
1944-1948	-0.13	0.84	0.63	1.3	-0.04	0.85	0.48	1.92
1949-1953	0	0.96	0.74	1.68	0.21	1.09	0.6	2.51
1954-1958	0	0.96	0.76	1.88	0.39	1.3	0.73	3
1959-1963	-0.1	0.87	0.71	1.49	0.13	1.01	0.53	2.46
1964-1968	-0.09	0.87	0.74	1.42	0.06	0.94	0.5	2.25
1969-1973	0.03	0.98	0.86	1.22	-0.07	0.82	0.45	1.92
1974-1978	0.17	1.13	1.02	1.02	-0.37	0.61	0.34	1.4
1979-1983	0.23	1.2	1.09	0.84	-0.62	0.48	0.26	1.11
1984-1988	0.25	1.23	1.12	0.9	-0.47	0.55	0.32	1.23
1989-1993	0.37	1.39	1.25	1.11	-0.28	0.67	0.38	1.51
1994-1998	0.34	1.35	1.17	1.88	0.17	1.05	0.56	2.53
1999-2003	0.08	1.03	0.81	3.63	0.83	2.02	0.97	5.37
2004-2009	0.01	0.95	0.49	9.87	1.07	2.57	0.63	7.33
**AIC**	5.96				4.76			
**BIC**	-163.54				-182.41			
**Deviance**	-163.54				9.29			

Notes: RR is Relative ratio [RR = exp.(coefficient)]; CI Confidence interval; Coef., coefficient; AIC, Akaike's information criterion; BIC, Bayesian information criterion.
